# Mitochondrial dysfunction in diabetic ulcers: pathophysiological mechanisms and targeted therapeutic strategies

**DOI:** 10.3389/fcell.2025.1625474

**Published:** 2025-08-21

**Authors:** Yu Pan, Lin Chen, Yan Chen, Elizabeth Rosalind Thomas, Shiying Zhou, You Yang, Kezhi Liu, Jianming Wu, Xiang Li

**Affiliations:** ^1^ Department of Biochemistry and Molecular Biology, School of Basic Medical Sciences, Southwest Medical University, Luzhou, China; ^2^ School of Nursing, Southwest Medical University, Luzhou, China; ^3^ Department of Dermatology, Department of Orthopedics and Joint Surgery, The Affiliated Hospital, Southwest Medical University, Luzhou, China; ^4^ Department of Medical Microbiology, PGIMER, Chandigarh, India; ^5^ Zigong Institute of Brain Science, Zigong mental health Center, The Zigong Affiliated of Hospital of Southwest Medical University, Zigong, Sichuan, China; ^6^ Health Science Center, Xi’an Jiaotong University, Xi’an, China

**Keywords:** diabetes, mitochondria, trauma, apoptosis, ROS

## Abstract

Diabetic foot ulcers (DFUs) are a serious complication of diabetes, characterized by delayed wound healing, recurrent infection, and risk of amputation. Mitochondrial dysfunction has emerged as a central pathological mechanism underlying impaired wound healing. Persistent hyperglycemia triggers a cascade of mitochondrial abnormalities like disrupted calcium homeostasis, excessive ROS production, impaired autophagy, increased apoptosis, and imbalanced mitochondrial dynamics. These alterations hinder ATP production, damage repair cells and delays tissue regeneration. This review comprehensively explores the mechanism of action of oxidative stress, mitochondrial apoptosis, autophagy dysfunction, calcium imbalance and ferroptosis on DFU pathogenesis. It also highlights promising mitochondrial targeted therapies. As mitochondria regulates key cellular processes, targeting mitochondrial dysfunction represents a novel and promising strategy. Future research should focus on integrated approaches to restore mitochondrial homeostasis in diabetic wound healing.

## 1 Introduction

Diabetes mellitus is a serious and chronic metabolic disease, characterized primarily by hyperglycemia. Its global incidence continues to rise, posing significant public health challenges. Among the numerous complications associated with diabetes, diabetic foot ulcers (DFUs) is one of the most severe and prevalent issue, affecting approximately 18.6 million individuals worldwide annually (Armstrong et al., 2023). The pathogenesis of DFUs is multifactorial, involving a complex interplay of pathological factors that ultimately result in cellular dysfunction and impaired wound healing. A critical aspect of diabetic ulcers is the altered wound microenvironment, particularly abnormalities in the extracellular matrix, which directly impair wound repair mechanisms (Santarella et al., 2020; Chang and Nguyen, 2021). These alterations manifest through disturbances in the immune microenvironment (Zhao et al., 2023; Mohsin et al., 2024), imbalances in cytokines (Zubair and Ahmad, 2019), growth factors (Yan et al., 2018; Zhang et al., 2023b), and dysregulated protease activity (McCarty et al., 2012; Gao et al., 2015; Chen et al., 2023b). Collectively, these factors disrupt the normal cellular functions, making wound healing exceedingly challenging. In addition, prolonged hyperglycemia contributes to serious neurological and vascular complications, diminishing sensory acuity and increasing vulnerability to skin injuries. Even minor lesions may serve as entry points for pathogenic microorganisms, gradually developing into chronic, hard-to-heal ulcers within the glucose-rich microenvironment of diabetic patients (Uberoi et al., 2024; Yang et al., 2024). Such ulcers significantly reduce patients’ quality of life, escalating healthcare costs, and can lead to severe outcomes such as infections, amputations (Schwarz et al., 2013), and even death (Senneville et al., 2024). Therefore, early diagnosis, timely intervention, and exploration of innovative treatment options are essential to improve outcome for patients with diabetic ulcers (McDermott et al., 2023).

Mitochondria, a primary site of cellular energy production and metabolism, play a pivotal role in maintaining cellular homeostasis (Spinelli and Haigis, 2018). Glucose undergoes glycolysis in the cytoplasm, generating pyruvate, which is converted to acetyl coenzyme A and enters the mitochondrial matrix to fuel oxidative phosphorylation. This process produces essential molecules like nicotinamide adenine dinucleotide (NADH) and flavin adenine dinucleotide (FADH_2_), which drives the electron transport chain, culminating in ATP generation (Walsh et al., 2018). Beyond energy metabolism, mitochondria are integral to diverse cellular processes, including intracellular calcium homeostasis (Cartes-Saavedra et al., 2025), reactive oxygen species (ROS) production (Zorov et al., 2014; Rizwan et al., 2020), regulation of intracellular protein folding (Shin et al., 2021), apoptosis, immune response modulation (Zecchini et al., 2023; Trinchese et al., 2024), mitochondrial quality control (Al Ojaimi et al., 2022), and mitophagy (Lorentzen et al., 2025).

Despite advances in conventional treatments, diabetic wounds often exhibit suboptimal healing. Targeting mitochondrial biological functions present a promising therapeutic approach. By improving cellular energy metabolism, reducing oxidative stress, promoting angiogenesis, inhibiting apoptosis, and modulating immune responses, mitochondrial-targeted interventions can accelerate tissue repair and wound healing (Lin M. et al., 2023; Qi et al., 2024). Consequently, improving mitochondrial function represents an urgent and promising strategy for the treatment of diabetic wounds.

Recent studies increasingly highlight the critical role of mitochondria in the pathogenesis and progression of diabetic ulcers (Jiang et al., 2023). This review summarizes the current status of mitochondrial function in diabetic ulcer healing and explores potential therapeutic approaches, providing a foundation for the development of improved clinical treatment strategies.

### 1.1 Effects of mitochondrial oxidative stress on diabetic wound healing

Under normal physiological conditions, wound healing consists of four successive stages: hemostasis, inflammation, proliferation, and remodeling (Lindley et al., 2016). However, in diabetic patients, this process is often disrupted by cellular dysfunction (Liu C. et al., 2023) and prolonged inflammation (Xia et al., 2023) that prevents progression to subsequent healing stages (Yang et al., 2023; Xiong et al., 2025). A prolonged hyperglycemic state in diabetic patients significantly increases the production of ROS (Yaribeygi et al., 2019b; Wan et al., 2022), including superoxide anion, hydrogen peroxide and hydroxyl radicals (Staveness et al., 2016). Under normal physiological conditions, ROS act as essential signaling molecules, regulates pathogen defense, autophagy and cell proliferation (Carrasco et al., 2015; Sahin et al., 2019; Li W. et al., 2024; Zhao et al., 2024).

However, in diabetic patients, the overproduction of ROS triggers oxidative stress, causing damage to cell membranes, proteins, and DNA, thereby impairing wound healing (Deng et al., 2021; Hong et al., 2023). Additionally, mitochondrial antioxidant defense systems - such as manganese superoxide dismutase (MnSOD), glutathione peroxidase (GPX), and glutathione reductase - exhibit diminished activity in diabetic patients (Oyewole and Birch-Machin, 2015; Li F. et al., 2024). This imbalance between ROS production and antioxidant capacity exacerbates tissue injury, disrupts cellular redox homeostasis, and further impairs healing (Yaribeygi et al., 2019a; Zhu et al., 2024). Notably, reduced peroxidase III expression in diabetic wounds is associated with mitochondrial membrane potential (ΔΨm), a key factor in triggering apoptotic signaling (Wolf et al., 2010; Wang X. et al., 2020; Wang et al., 2022). Consequently, mitochondrial dysfunction and ROS are recognized not only as markers but also as drivers of impaired healing in diabetic wounds (Wang X. et al., 2020).

**FIGURE 1:**
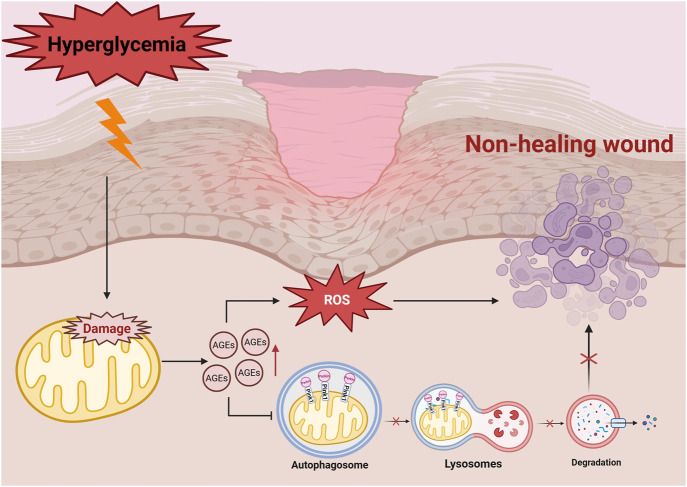
Hyperglycemia-affected cell with high ROS production triggers mitochondrial dysfunction, exacerbates oxidative stress, promotes the formation of AGEs, and further activates signalling pathways, such as NF-κB and PKC, which ultimately leads to delayed wound healing. Hyperglycemia induces the accumulation of AGEs, leading to increased levels of ROS, which in turn downregulates mitochondrial autophagy proteins such as PINK1/Parkin. As the autophagosome cannot fuse with the lysosome properly, mitochondrial degradation is blocked, which ultimately leads to non-healing wound in diabetes mellitus. Damaged mitochondria are not effectively cleared, which further exacerbates oxidative stress and disrupts cellular metabolism.

High levels of ROS also damages extracellular matrix (ECM) proteins, leading to non-enzymatic glycosylation due to excess glucose (Du et al., 2020; Sabbatinelli et al., 2022). This process generates intermediates that result in the formation of advanced glycation end-products (AGEs), which interacts with their corresponding receptors (RAGE), further accelerating glycosylation (Zheng et al., 2022) and exacerbating vascular and neural toxicity, while impairing the functions of macrophages (Geng et al., 2023), fibroblasts, and vascular endothelial cells (Mao et al., 2022) - all of which are critical for wound healing (Li et al., 2022b; Fu et al., 2023). Moreover, diabetic patients also exhibit reduced antioxidant enzyme activity in the ECM, making their wounds highly sensitive to oxidative stress, particularly during the tissue remodeling phase (Kunkemoeller and Kyriakides, 2017; Elbatreek et al., 2019). The activation of the AGE-RAGE signaling pathway is a key component in driving the vicious cycle of oxidative stress (Shi et al., 2013). The binding of AGE-RAGE activates NADPH oxidase (NOX), leading to the generation of large amounts of cytoplasmic ROS (Chen et al., 2018), and NOX-derived ROS contributes to mitochondrial dysfunction, secondary ROS generation, mtDNA damage, and impaired antioxidant defenses. Continuous activation of the AGE-RAGE pathway leads to the accumulation of ROS, which impedes the healing of diabetic wounds (Piperi et al., 2015; Bai et al., 2022; Zhang et al., 2025) (Figure 1).

In addition, oxidative signals activates multiple signaling pathways, such as protein kinase C (PKC), nuclear factor-κB (NF-κB), mitogen-activated protein kinase (MAPK), and c-Jun N-terminal kinase/stress-activated protein kinase (JNK/SAPK) (Choudhury et al., 2015; Qin et al., 2019; Meng W. et al., 2025). Activating these signaling pathways regulates matrix metalloproteinase expression, and combined with high levels of redox reactions, disrupts ECM remodeling, delays wound healing, and promotes inflammation and apoptosis (Kowluru et al., 2016; Zhu et al., 2022). It is worth noting that damage-associated molecular patterns (DAMPs) (e.g., mtDNA, ATP) released by mitochondrial dysfunction can trigger sterile inflammation through TLRs, NLRP3 inflammasomes, and cGAS-STING pathways, hindering diabetic wound healing (Grazioli and Pugin, 2018). Improving mitochondrial function by suppressing mtDNA leakage and promoting ATP production reduce inflammatory responses and accelerate wound healing (He et al., 2024; Mao et al., 2025).

A prolonged hyperglycemic state significantly increases electron leakage in complexes I and III of the electron transport chain (ETC), generating large amounts of superoxide anions (O_2_^−^) (Kowluru et al., 2016; Huo et al., 2023). These anions get converted into hydroxyl radicals (OH) and hydrogen peroxide (H_2_O_2_), which damage the mitochondrial structures, reducing ATP production, decreasing ΔΨm, and induces mitochondrial DNA (mtDNA) mutations (Yuzefovych et al., 2012; Li et al., 2022a; Shen et al., 2023). Studies have shown that introducing the 8-oxo guanine DNA glycosylase 1 gene into mitochondria using adenoviral vector technology effectively mitigates mtDNA damage, thereby improving ROS and promoting wound-healing in diabetic rat models (Yuzefovych et al., 2012).

Furthermore, Rizwan et al. found that excess ROS in hyperglycemic environments damages mtDNA in keratinocytes (Rizwan et al., 2020), triggering inflammation and apoptosis via the cGAS-STING-IRF3 pathway. Targeting mtDNA protection offers a great potential strategy for diabetic wound healing. Chronic hyperglycemia also suppresses vascular regeneration, prolongs epithelial migration, exacerbates inflammatory cell infiltration, and hinders granulation tissue formation, all of which contribute to poor wound healing (Peplow and Baxter, 2012; Luo et al., 2023). Prolonged exposure of the body to high glucose levels activates hypoxia-induced pathways, further perpetuating inflammation and tissue damage (Gerber and Rutter, 2017; Huang et al., 2024). However, Shi et al. revealed that bone marrow mesenchymal stem cells (BMSCs) under hypoxic conditions secretes TGF-β1, promotes autophagy, reduces inflammation, and enhancing epidermal cell proliferation and migration through the HIF-1α/TGF-β1/SMAD signaling pathway. This accelerated wound healing in diabetic ulcers (Shi et al., 2022). These findings suggest that hypoxia, despite its detrimental role in diabetic wounds, may have context-dependent therapeutic potential warranting further investigation.

ROS exhibit a dual role in wound healing; while moderate ROS levels stimulate early inflammatory responses, recruit immune cells and promote angiogenesis and epithelial migration (Sies and Jones, 2020), excessive ROS induces oxidative stress, damages cellular structures (Li M. et al., 2024), and activates pro-inflammatory pathways, such as NF-κB, impeding healing (Ji et al., 2024).

Novel approaches, like the glucose-responsive hydrogel GHM3, show promise. GHM3 reduces glucose levels in the wound microenvironment, scavenges ROS, improving inflammation and accelerating wound healing (Qi et al., 2023).

In summary, mitochondrial dysfunction and oxidative stress play crucial roles in the pathophysiology of diabetic wound healing. Strategies to inhibit ROS overproduction, maintain mitochondrial function, and enhance antioxidant defenses are essential for improving wound healing in diabetic patients.

### 1.2 Effects of mitophagy on diabetic wound healing

Mitophagy is a self-regulatory mechanism that maintains cellular homeostasis by selectively removing damaged or dysfunctional mitochondria (Jiang et al., 2023). It plays a crucial role in regulating cellular metabolism and stress responses. In diabetic patients, the hyperglycemic microenvironment and the subsequent accumulation of AGEs impair cellular mitophagy, delay wound healing and disrupts cellular metabolism (Han et al., 2017; Wu et al., 2024). Studies have shown that mitophagy - related proteins, such as PINK1, Parkin, Beclin1 and LC3-II/LC3-I are significantly downregulated during the wound infection stage, exacerbating mitochondrial dysfunction as the condition progresses (Xiang et al., 2022; Deng et al., 2024).

Angiogenesis is critical for wound healing during the proliferation phase, and mitophagy promotes vascular endothelial cell survival and proliferation, facilitating the formation of new blood vessels (Zhu et al., 2018; Fan et al., 2023). Laughlin et al. demonstrated that enhancing the level of mitophagy in keratinocytes counteracted the negative effects of AGEs, promoting differentiation, proliferation, and epithelialization in diabetic ulcers (Laughlin et al., 2020). However, in diabetic patients, oxidative stress and hyperglycemia inhibits mitophagy, impairs angiogenesis and hinders wound repair. Collectively, mitophagy dysfunction emerges as a key factor contributing to impaired wound healing in diabetes.

Mitophagy reduces ROS accumulation by efficiently removing damaged mitochondria, thus lowering oxidative stress and maintaining normal cellular metabolism–crucial for wound healing (Tan et al., 2022; Zhao et al., 2022). Interestingly, ROS also play a dual role in mitophagy: excessive oxidative stress impairs mitophagy, while low levels activate mitophagy as a protective response (Liu J. et al., 2023).

The PINK1/Parkin pathway is a fundamental regulatory mechanism in mitophagy (Figure 1) (Wang H. et al., 2024). In healthy cells, PTEN-induced kinase 1 (PINK1) is imported into the mitochondria via the translocase complexes (TOM and TIM) and is rapidly degraded (Eldeeb et al., 2024). However, under oxidative stress, mitochondrial depolarisation prevents PINK1 degradation, enabling it to recruit Parkin to label damaged mitochondria for autophagic clearance (Shu et al., 2021). The damaged mitochondria are degraded by lysosomal enzymes into essential biomolecules such as amino acids and lipids, which are reused for cell regeneration and tissue repair (Zhang et al., 2022). In diabetic wounds, impaired mitophagy reduces the metabolic recycling capacity. Further research is needed to elucidate its exact role in delayed diabetic wound healing (Figure 1).

Mitophagy reduces the release of apoptotic factors, thereby preserving tissue integrity and cellular function. Studies show that mitophagy related proteins, including PINK1, Parkin, LC3-I and Beclin1, are downregulated in vascular endothelial cells under hyperglycemic conditions (Xi et al., 2021; Xiang et al., 2022), resulting in mitochondrial damage, increased apoptosis, and reduced endothelial cell activity and migration (Figure 1). Su et al. demonstrated that denatured collagen enhances autophagy and inhibits fibroblast apoptosis, facilitating wound repair (Su et al., 2022). In fibroblasts, denatured collagen reduces the activation of the apoptotic marker, caspase-3 and increases the expression of autophagic markers like Beclin-1 and LC3, highlighting its potential in promoting diabetic wound healing.

Chen et al. further confirmed the critical role of mitophagy in diabetic wound healing (Chen et al., 2015), showing that high glucose levels inhibit autophagy in endothelial progenitor cells (EPCs), increasing apoptosis and impairing its function. It is well known that mechanistic target of rapamycin (mTOR) plays an important role in the regulation of autophagy. Inhibiting mTOR signaling pathway activity reverses AGEs-induced autophagy impairment in endothelial progenitor cells (EPCs), thereby accelerating wound healing in diabetes (Jin et al., 2018). These findings underscore the importance of regulating mitochondrial mitophagy to reduce mitochondrial damage, inhibit apoptosis, and improve diabetic wound healing.

The imbalance between autophagy and apoptosis is a major contributor to delayed healing in diabetic wounds (Ko et al., 2020). Regulating autophagy pathways and addressing mitochondrial dysfunction could significantly enhance therapeutic strategies. Future research should focus on elucidating the precise mechanisms of autophagy inhibition in diabetic wounds, identifying novel targets and developing treatments to restore cellular homeostasis and accelerate wound healing.

### 1.3 Mitochondrial fission and fusion in diabetic wound healing

Mitochondrial quality control is a dynamic process through which mitochondria regulate their morphology, size, number, and function to maintain cellular homeostasis, respond to oxidative stress, and regulate energy metabolism (Jiang et al., 2022). Mitochondrial fission and fusion - the two key dynamics of mitochondrial quality control - enable mitochondria to adapt to cellular demands, ensuring the proper balance required for cellular function and environmental adaptation (Liu B. H. et al., 2024). Disruption of this balance is implicated in various pathological conditions, including diabetic wound healing (Zheng et al., 2021).

Mitochondrial quality control processes are precisely regulated by a specific set of regulatory proteins. During mitochondrial fission, the primary regulatory proteins include dynamin-related protein 1 (Drp1) and its receptor proteins - mitochondrial fission protein 1 (Fis1), mitochondrial fission factor (MFF) and MiD49/MiD51 (Konig et al., 2021). Drp1, a GTPase, is recruited to the outer mitochondrial membrane where it forms oligomeric structures with these receptor proteins (Gao and Hu, 2021), thus playing a central role in mitochondrial constriction and division. This process ensures proper mitochondrial distribution during cell division and the removal of damaged mitochondria (Kleele et al., 2021).

**FIGURE 2:**
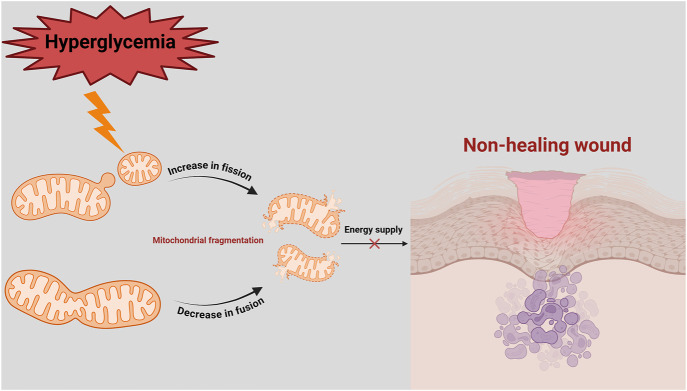
Imbalance of mitochondrial fission and fusion in delayed diabetic wound healing. In a hyperglycaemic environment, mitochondrial fission is increased, while mitochondrial fusion is decreased. This leads to mitochondrial fragmentation, energy deficit (decreased ATP) and ROS production. Since diabetic wounds have a high energy demand, inadequate ATP supply and imbalance in mitochondrial quality control lead to prolonged non-healing of diabetic wounds.

In diabetic patients, chronic hyperglycemia and impaired insulin signaling increases cellular energy demand (Tseng et al., 2024). To compensate, mitochondrial fission proteins are upregulated, while mitochondrial fusion proteins are downregulated. However, excessive activation of Drp1 leads to overactive mitochondrial fission, producing dysfunctional mitochondria that produces large amounts of ROS (Hao et al., 2019). This exacerbates intracellular oxidative stress, creating a vicious cycle of mitochondrial damage and cellular dysfunction (Ruegsegger et al., 2018) (Figure 2). Zhang et al. demonstrated that high glucose conditions lead to rapid mitochondrial fragmentation and increased expression of fission-related proteins, such as Drp1 and Fis1, disrupting mitochondrial morphology and exacerbating ROS production (Zhang et al., 2023a). Shi et al. showed that inhibiting the ROCK1/Drp1 mediated mitochondrial fission pathway reduced mitochondrial ROS (mtROS) production, restored blood flow, promoted capillary formation, and accelerated wound healing in diabetic mice (Shi et al., 2018).

Conversely, mitochondrial fusion involves the progressive merging of two or more mitochondria into a continuous network, including both inner and outer mitochondrial membranes. This process is mediated primarily by mitochondrial fusion proteins such as optic atrophy 1 (OPA1), mitofusin1 (Mfn1) and mitofusin2 (Mfn2) (Hu et al., 2021). Mfn1 and Mfn2 mediates the fusion of outer mitochondrial membrane by forming homodimers or heterodimers (e.g., Mfn1-Mfn1, Mfn2-Mfn2, or Mfn1-Mfn2), while OPA1 mediates inner membrane fusion (Gordaliza-Alaguero et al., 2024). Mitochondrial fusion enhances bioenergetic capacity, supports mitochondrial genome intergrity, and enables cells to respond more effectively to injury and stress (Tabara et al., 2024). Lu et al. found that mesenchymal stem cells’ extracellular vesicles (MSC-EVs) promote mitochondrial fusion, reduce ROS and mtDNA release, and inhibit ferroptosis in endothelial cells, thus improving angiogenesis and wound healing in diabetic mice (Lu et al., 2024).

In diabetic patients, the balance between mitochondrial fission and fusion is often disrupted, as evidenced with increased fission and decreased fusion observed under hyperglycemic stress (Tatmatsu-Rocha et al., 2018). This imbalance contributes to mitochondrial fragmentation, functional impairment, and oxidative stress, all of which exacerbate endothelial cell dysfunction, apoptosis, and impaired wound healing (Sun et al., 2016). Zheng et al. reported that high-glucose induced dysregulation of mitochondrial dynamics disrupts vascular endothelial function, with upregulated fission proteins (Drp1 and Fis1) and downregulated fusion proteins (Mfn1, Mfn2, and OPA1), contributing to mitochondrial dysfunction and increased oxidative stress (Zheng et al., 2021) (Figure 2).

Mitochondrial fusion is particularly crucial for repairing damaged mitochondria and maintaining energy metabolism (Quintana-Cabrera et al., 2021; Guo et al., 2023). Disruption of fusion proteins impairs ATP production, directly affecting energy intensive processes such as cell migration, proliferation, and tissue regeneration, which are essential for wound healing (Amini et al., 2018; Sun et al., 2022). Wang et al. showed that overexpression of NDUFB5 promotes mitochondrial fusion, restores mitochondrial oxidative phosphorylation, and accelerates diabetic wound healing by improving mitochondrial function and reducing ROS production (Wang T. et al., 2024). Similarly, Chen et al. found that the S1PR2 antagonists modulate the RhoA/ROCK1/Drp1 signaling pathway, reversing high glucose-induced mitochondrial fission, improving endothelial cell migration, and inhibiting apoptosis (Chen et al., 2019).

Therefore, dysregulation of mitochondrial dynamics plays an essential pathological role in diabetic wound healing (Dai et al., 2022). An in-depth research is needed to elucidate the molecular mechanisms governing mitochondrial fission and fusion in diabetic wounds. Understanding these pathways can pave the way for new treatments that address mitochondrial dysfunction, providing innovative therapeutic avenues for managing diabetes-related complications.

### 1.4 Mitochondrial apoptosis in diabetic wound healing

Apoptosis, a genetically controlled, process of programmed cell death, plays a critical role in tissue homeostasis by rapidly removing excess or damaged cells. This process involves two major pathways: the mitochondria-mediated intrinsic pathway and the death receptor-mediated extrinsic pathway (Son and Lee, 2021). Among these, the mitochondria-mediated pathway is the predominant intrinsic apoptotic mechanism (Brenner and Mak, 2009). In DFUs, the activation of mitochondrial apoptosis disrupts physiological processes such as cell proliferation, angiogenesis, and reconstruction of extracellular matrix (Nagarjuna Reddy et al., 2022). The apoptosis of key cells, including fibroblasts, keratinocytes, and vascular endothelial cells, delays wound healing and hampers tissue regeneration (Kim and Park, 2019; Liang et al., 2019; Liu H. et al., 2024), highlighting the critical role of apoptotic mechanisms in wound healing.

Mitochondrial apoptosis is initiated by multiple pro-apoptotic signals, including oxidative stress triggered by hyperglycemia, sustained inflammation, DNA damage, and severe hypoxia (Kaina, 2003; Chen et al., 2024). These pro-apoptotic signals act synergistically through BH3 domain - containing proteins, such as Bim and Bid, which activates key pro-apoptotic effectors like Bax and Bak (Ruhl et al., 2025). When activated, Bax and Bak translocate to the outer mitochondrial membrane, resulting in mitochondrial outer membrane permeabilisation (MOMP) (Riley et al., 2018). This disrupts membrane integrity, decreases mitochondrial membrane potential, and facilitates the release of pro-apoptotic factors such as cytochrome C (Cyt C) (Cheng and Ferrell, 2018).

**FIGURE 3:**
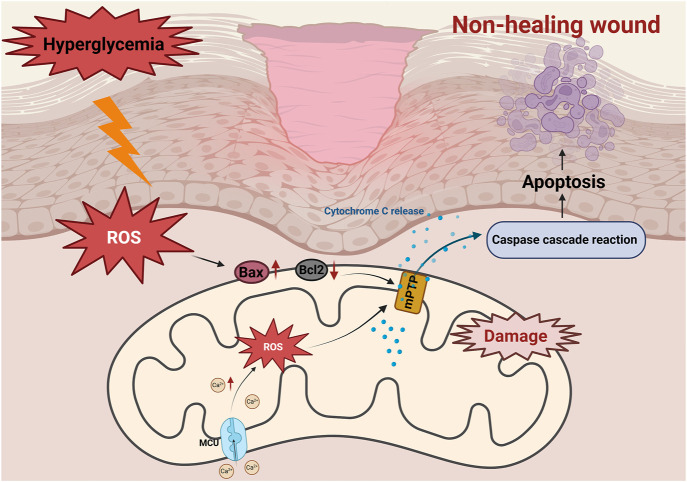
Role of mitochondrial apoptosis in non-healing diabetic wounds. Activation of the AGEs/RAGE axis induces overproduction of ROS, leading to upregulation of Bax and downregulation of Bcl-2. The subsequent opening of the mitochondrial membrane permeability transition pore (mPTP) triggers a decrease in ΔΨm, which leads to the release of Cyt C and activation of the caspase cascade reaction, ultimately inducing apoptosis of fibroblasts, exacerbating tissue repair disorders and delaying healing of diabetic wounds. Hyperglycemia triggers mitochondrial calcium (Ca^2+^) overload through MCU channels. Excess Ca^2+^ triggers ROS accumulation, disrupts mitochondrial membrane potential (ΔΨm), and causes Cyt C release, activates caspase cascade reaction, which ultimately induces apoptosis and leads to delayed diabetic wound healing.

In DFUs, hyperglycemia disrupts the balance between pro-apoptotic (e.g., Bax) and anti-apoptotic (e.g., Bcl-2) proteins, triggering mitochondrial apoptosis (Wu et al., 2025). Studies on diabetic wounds have show significant reduced expression of Bcl-2, and weakened anti-apoptotic defenses, leading to cell apoptosis (Ye et al., 2025) (Figure 3). This phenomenon exacerbates the loss of fibroblasts and vascular endothelial cells, impairs tissue regeneration and delays wound healing (Justynski et al., 2023). Moreover, AGEs activate apoptotic signaling pathways through interaction with their receptor, RAGE. This interaction enhances the production of ROS and upregulates the expression of Bax, caspase-9, and cytochrome c, ultimately activating apoptotic markers such as caspase-3 and PARP. Consequently, endothelial progenitor cell apoptosis occurs, impairing tissue repair and further delaying wound healing (Jin et al., 2018; Li et al., 2018). Similarly, Ren et al. found that hyperglycemia increases the expression of cleaved Bax and Caspase-3 in human microvascular endothelial cells (HMEC-1), promoting apoptosis, oxidative stress and inflammation. However, increased expression of angiotensin-converting enzyme 2 (ACE2) was found to attenuate hyperglycemia-triggered apoptosis by inhibiting the JAK2/STAT3 signaling pathway, improving cell viability, and decreasing mitochondrial apoptotic protein expression (Ren et al., 2022). This suggests that ACE2 could be a potential therapeutic target for improving vascular endothelial cell dysfunction and promoting wound healing in diabetic patients.

Changes in mitochondrial membrane permeability is pivotal in mitochondria-mediated apoptosis, directly determining cell fate (Tomasina et al., 2022). Increased mitochondrial membrane permeability facilitates the rapid release of Cyt C, a key mediator in the mitochondrial respiratory chain. Cyt C disrupts electron transfer, impairs respiratory chain function, and leads to excessive production of superoxide ions. This triggers oxidative stress, exacerbates the inflammatory response and further delays wound healing in diabetic patients (Figure 3).

In addition, extrinsic apoptosis plays an important role in wound healing in diabetes. TNF-α inhibition promoted wound healing in diabetic mice and inhibited fibroblast apoptosis (Siqueira et al., 2010). In diabetic wound, TNF superfamily, member 6 (FasL) expression induces keratinocyte apoptosis, leading to delayed healing (Liang et al., 2019; Wang et al., 2023). Modulating mitochondrial apoptotic proteins or directly targeting mitochondrial function presents a promising intervention strategy for diabetic wound management. However, the precise mechanisms by which mitochondrial apoptosis influences diabetic wound healing remains incompletely understood. Further research is needed to explore the molecular pathways involved and develop targeted pharmacological interventions. Advancing our understanding in this area could open new avenues for the treatment of diabetes-induced complications and accelerate wound repair.

### 1.5 Mitochondrial calcium homeostasis and its role in diabetic wound healing

Mitochondria, often referred as the “powerhouse” of the cell, serves as an important regulatory centre for intracellular calcium signaling (De Stefani et al., 2016). In 1960, De Luca et al. first discovered that mitochondrial calcium uptake is mediated by the mitochondrial calcium uniporter (MCU), a channel located across the inner mitochondrial membrane. The MCU complex consists of channel subunits (MCU and MCUb), regulatory subunits (EMRE, MICU1, MICU2, and MCUR1), and additional proteins associated with calcium transport. Calcium ion (Ca^2+^) translocation is regulated by the interaction between the channel subunits and the regulatory proteins (Xue et al., 2022).

The mitochondrial Ca^2+^ uptake is driven by the electrochemical gradient established during oxidative phosphorylation, where the proton concentration gradient across the inner mitochondrial membrane fuels ATP synthesis (Patron et al., 2022; Szabo and Szewczyk, 2023). This Ca^2+^ influx is essential for regulating aerobic metabolism and maintaining redox homeostasis (Wescott et al., 2019). However, disrupted mitochondrial calcium homeostasis triggers oxidative stress, overproduction of ROS, mitochondrial depolarization, and apoptosis (Meng et al., 2023; Weiser et al., 2023).

In the resting state, the cytoplasmic Ca^2+^ concentration is low, and regulatory proteins such as MICU1 and MICU2 prevent Ca^2+^ from entering the mitochondria through MCU (Liu et al., 2016). Upon stimulated, cytoplasmic Ca^2+^ levels rises and activates MICU1, enabling mitochondrial Ca^2+^influx; closure of MCU, mediated by MCUR1, restores balance (Dong et al., 2017). After sufficient calcium uptake, MCUR1 assists in closing the MCU channel, preventing calcium overload. This tightly regulated mechanism ensures that the mitochondria is protected from oxidative damage caused by excess calcium while maintaining their ability to respond to cytoplasmic calcium signals, thereby preserving normal physiological functions of the cells (Wang C. et al., 2020; Garbincius and Elrod, 2022).

Chronic hyperglycemic induces persistent oxidative stress, which disrupts mitochondrial calcium homeostasis (Gerber and Rutter, 2017). Normally Ca^2+^ helps regulate NADPH production to counteract oxidative stress (Park et al., 2022). However, in diabetes, mitochondrial dysfunction leads to calcium dysregulation, impairing the mitochondrial electron transport chain and decreasing the membrane potential (Belosludtsev et al., 2019; Dia et al., 2020), which exacerbates ROS generation and delays wound healing.

Mitochondrial calcium plays a dual role: in moderate amounts, it activates enzymes in the tricarboxylic acid (TCA) cycle, promoting ATP production. However, ROS-induced damage to the mitochondrial membrane causes excess calcium influx into the mitochondria, disrupts this balance. Chen et al. showed that MCU’s mRNA and its regulatory protein MCUR1 were upregulated in high-glucose environments, leading to increased mitochondrial calcium levels and ROS production. This, in turn, triggers endothelial cell dysfunction, apoptosis and impaired wound healing (Chen et al., 2017). Regulating MCU expression improves Ca^2+^ homeostasis, thereby protecting the biological function of dermal fibroblasts in wound healing (Wang M. et al., 2024).

Calcium overload opens the mitochondrial permeability transition pore (mPTP), a critical event in mitochondrial apoptosis (Dubois et al., 2024). The mPTP opening leads to a loss of mitochondrial membrane potential, mitochondrial swelling, and the release of pro-apoptotic factors such as Cyt C. These events exacerbate apoptosis, further impairing diabetic wound healing (Figure 3).

Therefore, hyperglycemia-induced oxidative stress and dysregulation of mitochondrial calcium homeostasis form a self-reinforcing loop. Oxidative stress damages mitochondrial membrane and impairs MCU function, leading to Ca^2+^ overload (Zhang et al., 2024). The imbalance increases ROS production, aggravating oxidative stress (Ly et al., 2017). This vicious cycle ultimately delays wound healing in diabetic patients. Although these mechanisms are theoretically supported, the exact processes remains to be determined.

Future studies should aim to further investigate the molecular pathways involved and develop new strategies to effectively regulate mitochondrial calcium homeostasis as a means to treat diabetic wounds.

### 1.6 Ferroptosis and its role in diabetic wound healing

Mitochondrial dysfunction is an important factor contributing to delayed wound healing in diabetic ulcers. Studies have shown that mitochondrial dysfunction is strongly associated with ferroptosis, a hallmark of diabetes-related complications (He et al., 2022). Ferroptosis, a Fe^2+^-dependent type of programmed cell death, differs from traditional modes of cell death such as apoptosis, necrosis, and autophagy. It is characterized by excessive lipid peroxidation. Mitochondria, enriched in Fe^2+^, serves as the primary site of ROS production, which enhances cellular sensitivity to ferroptosis through a variety of mechanisms.

**FIGURE 4:**
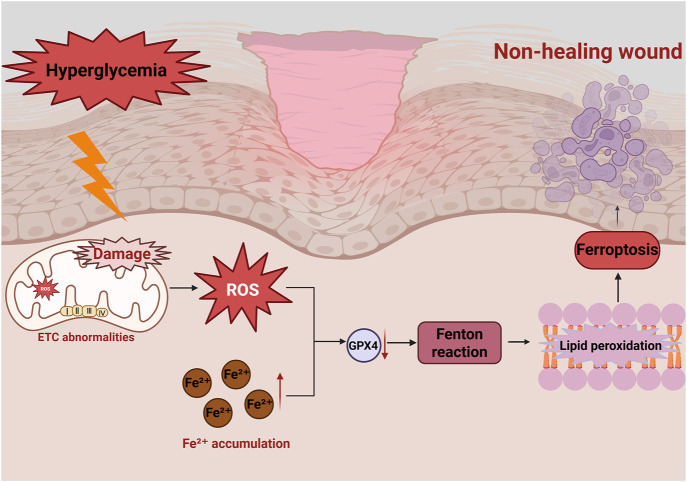
Role of ferroptosis in diabetic wounds in non-healing diabetic wounds. Hyperglycemia induces mitochondrial dysfunction, leading to excessive ROS production, reduced GPX4 activity and ETC., abnormalities. Excessive Fe^2+^ accumulation induces the Fenton reaction to produce lipid peroxides, which results in ferroptosis. This damage repair cells and impede wound healing.

In diabetic conditions, chronic hyperglycemia induces persistent oxidative stress, leading to excessive ROS production and Fe^2+^ accumulation (Wei et al., 2020; Feng et al., 2021). This activates lipid peroxidation, impairs cellular functions, and triggers ferroptosis, ultimately delaying wound healing (Feng et al., 2022) (Figure 4). However, the mechanisms linking mitochondrial dysfunction to ferroptosis in diabetic wounds remains incompletely understood, warranting further studies.

The critical ferroptosis inhibitors are glutathione peroxidase 4 (GPX4) and glutathione (GSH) (Xi et al., 2025). Their depletion enhances lipid peroxide accumulation, exacerbating ferroptosis, leading to cellular damage and delayed tissue repair (Li et al., 2021).

Hyperglycemia-induced mitochondrial dysfunction and endoplasmic reticulum stress can result in Fe^2+^ accumulation and excessive ROS production, further driving ferroptosis (Ma et al., 2020). Cui et al. found that elevated Fe^2+^ levels and ROS production damage mitochondria, impairing the proliferation and migration of critical skin repair cells such as human dermal fibroblasts (HDFs) and endothelial cells (Cui et al., 2023), significantly hindering diabetic wound healing. Studies have shown that administration of the Deferoxamine (DFO) improves ferroptosis in human umbilical vein endothelial cells (HUVECs) induced by high glucose (Chen et al., 2023a).

Xiong et al. (2024) developed a novel therapeutic approach using a PF-PEG@ASIV-EXO hydrogel, which inhibited ferroptosis pathways to promote wound healing. The hydrogel improved mitochondrial function, inhibited ferroptosis, and promoted angiogenesis by increasing the expression of SLC7A11, GPX4, mitochondrial GSH and superoxide dismutase (SOD), while decreasing the expression of the Acyl-CoA synthetase long chain family member 4 (ACSL4) – accelerating wound healing. This finding underscores the therapeutic potential of ferroptosis inhibitors in diabetic wound therapy.

Thus, hyperglycemia-induced ferroptosis contributes significantly to diabetic ulcer pathology by altering mitochondrial function, increasing oxidative stress and promoting lipid peroxidation (Meng X. et al., 2025). While the interplay between mitochondrial dysfunction and ferroptosis in diabetic ulcers is still unclear, targeting ferroptosis-related pathways holds significant therapeutic promise. Ferroptosis inhibitors, along with strategies to enhance mitochondrial health and regulate oxidative stress, could accelerate the healing of diabetic ulcers.

Thus, the modulation of ferroptosis pathways not only provides insights into pathological mechanism of diabetic ulcers but also offers a foundation for developing novel therapeutic strategy for future clinical interventions (Han et al., 2025).

## 2 Conclusion

**FIGURE 5:**
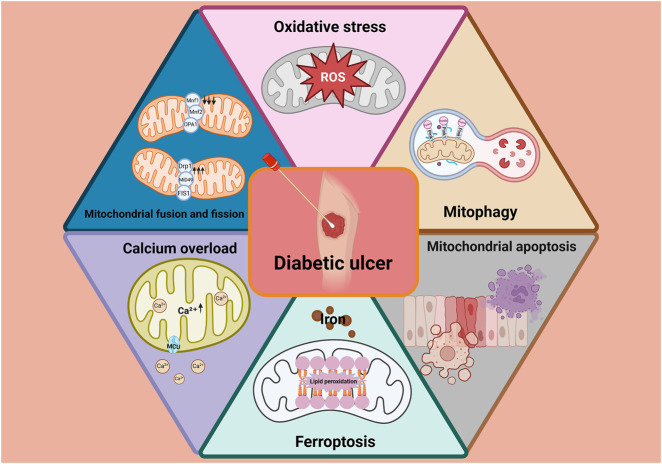
Sustained hyperglycemia-induced mitochondrial dysfunction–characterized by disrupted calcium homeostasis, excessive ROS production, impaired mitophagy, increased apoptosis and ferroptosis, and altered mitochondrial dynamics–is a central pathological mechanism hindering diabetic wound healing.

Sustained hyperglycemia-induced mitochondrial dysfunction–characterized by disrupted calcium homeostasis, excessive ROS production, impaired mitophagy, increased apoptosis and ferroptosis, and altered mitochondrial dynamics–is a central pathological mechanism hindering diabetic wound healing (Figure 5). These dysfunctions impair cellular energy metabolism and compromise the activity of critical repair cells, leading to delayed tissue regeneration. Consequently, therapeutic strategies aimed at restoring mitochondrial function–particularly by modulating calcium signaling, mitochondrial dynamics, mitophagy, and ferroptosis–hold significant promise.

Many drugs have shown to have better therapeutic effects on diabetes (Lin Y. et al., 2023; Oladoja et al., 2023). Notably, mitochondrial modulators such as metformin have demonstrated beneficial effects in diabetic wound models. Metformin inhibits excessive mitochondrial fission, reduces oxidative stress, and enhances mitophagy, collectively promoting wound repair. Table 1 summarizes pharmacological agents that target mitochondrial pathways for diabetic wound treatment.

**TABLE 1:** Drugs targeting mitochondria improve diabetic wound healing.

Drug	Mechanisms	Target	Outcome	References
Negative ion	Providing antioxidant, anti-inflammatory, anti-apoptotic effects; promoting angiogenesis	Mitochondrial autophagy and inflammation	Promoting diabetic wound healing	Cheng et al. (2022)
Photobiomodulation (laser/LEDs)	Regulating mitochondrial fusion and fission; promoting collagen production	Mitochondrial quality control	Enhancing collagen production and angiogenesis in diabetic wounds	Tatmatsu-Rocha et al. (2018)
Lonicerin	Promoting angiogenesis through Sirt1-mediated autophagy	Mitochondrial Sirt1 autophagy	Improving diabetic wound healing and angiogenesis	Lin et al. (2024)
Vildagliptin	Inhibiting Drp1 mediated mitochondrial division; ameliorating high glucose-induced mitochondrial dysfunction	Mitochondrial quality control	Protecting endothelial cell mitochondrial function and promoting diabetic wound healing	Liu et al. (2019)
Metformin	Enhancing autophagy through the AMPK activation; regulating HIF-1α levels	Mitochondrial autophagy	Activating AMPK and autophagy, improving blood vessel formation and promoting wound healing	Tombulturk et al. (2024)
Calcium channel modulator	Regulating mitochondrial calcium overload; inhibiting apoptosis	Mitochondrial homeostasis, apoptosis	Reducing oxidative stress and apoptosis; promoting angiogenesis in hyperglycemic environements	Chen et al. (2017)
Hesperetin	Activating SIRT3; inhibiting cellular ferroptosis	Ferroptosis, mitochondrial function	Inhibiting ferroptosis and promoting wound healing	Yu et al. (2024)
Exosome/Metformin Hydrogel	Inhibiting Drp1 mediated mitochondrial division; promoting wound healing and microvascular recovery	Mitochondrial quality control	Improving chronic wound healing and restoring microvascular function in diabetes mellitus	Zhang et al. (2023a)
Resveratrol	Regulating Nrf2 pathway; inhibiting ferroptosis; promoting angiogenesis	Ferroptosis	Enhancing diabetic wound healing and angiogenesis	Xiao et al. (2024)
Crocetin	Inhibiting AGEs-induced apoptosis; providing antioxidant effects; stabilizing calcium homeostasis	Mitochondrial oxidative stress, calcium homeostasis	Preventing vascular complications and protecting vascular endothelial cells during diabetes	Xiang et al. (2006)
Platelet-rich plasma	Inhibiting ferroptosis; promoting vascular regeneration; reducing oxidative stress and inflammation	Mitochondrial oxidative stress, ferroptosis	Promoting healing of type 2 diabetic ulcers and restoring vascular endothelial cell function	Chen et al. (2022)

Future research should focus on developing integrated therapeutic approaches that comprehensively regulate mitochondrial biological functions. Such strategies offer a targeted and effective path toward improved clinical outcomes in diabetic wound healing.
